# Effect of Adiponectin on the Expression of Selected Cytokines in Periodontal Ligament Cells

**DOI:** 10.3390/biology14040321

**Published:** 2025-03-21

**Authors:** Małgorzata Kozak, Agata Poniewierska-Baran, Michał Czerewaty, Karolina Łuczkowska, Krzysztof Safranow, Małgorzata Mazurek-Mochol, Bogusław Machaliński, Andrzej Pawlik

**Affiliations:** 1 Department of Dental Prosthetics, Pomeranian Medical University, 70-111 Szczecin, Poland; gosia-ko@o2.pl; 2 Institute of Biology, University of Szczecin, 71-412 Szczecin, Poland; agata.poniewierska-baran@usz.edu.pl; 3 Department of Physiology, Pomeranian Medical University, 70-111 Szczecin, Poland; michal.czerewaty@pum.edu.pl; 4 Department of General Pathology, Pomeranian Medical University, 70-111 Szczecin, Poland; karolina.luczkowska@pum.edu.pl (K.Ł.); machalin@pum.edu.pl (B.M.); 5 Department of Biochemistry and Medical Chemistry, Pomeranian Medical University, 70-111 Szczecin, Poland; chrissaf@mp.pl; 6 Department of Periodontology, Pomeranian Medical University, 70-111 Szczecin, Poland; malgorzata.mazurek@poczta.onet.pl

**Keywords:** adiponectin, periodontal ligament, cytokines

## Abstract

Periodontitis is a disease caused by a bacterial infection that causes chronic inflammation. The pathogenesis of periodontitis is mediated by several mediators, including chemokines, cytokines, metalloproteinases, and adipokines. Adiponectin is an adipokine that influences several metabolic processes and numerous immunological processes. In this study, we investigated the effect of adiponectin on the expression in the periodontal ligament of selected cytokines involved in the pathogenesis of periodontitis. Human periodontal ligament cells (hPDLCs) were stimulated with adiponectin and then analyzed for expression (TNF-α, IL-1, IL-6, IL-8, IL-10, IL-17, and IL-18) in cell cultures at the mRNA level and in supernatants at the protein level. The samples were analyzed after 12, 24, and 48 h of adiponectin stimulation. We found no significant effect of adipokine on TNF-α gene expression after 12, 24, and 48 h of stimulation. For IL-1, a statistically significant increase in *IL-1* gene expression was found after 12 h of adiponectin stimulation, while the differences were not statistically significant after 24 and 48 h. Adiponectin caused a statistically significant increase in *IL-6* gene expression after 12, 24, and 48 h of stimulation. Stimulating periodontal ligament cells with adiponectin significantly increased TNF-α, IL-6, and IL-8 protein levels in supernatants after 12, 24, and 48 h. The levels of IL-1 were statistically significantly increased after 12 and 24 h of adiponectin stimulation. There was no statistically significant effect of adiponectin on IL-10, IL-17, and IL-18 levels. The results of our study suggest that adiponectin may significantly increase the expression of selected cytokines in periodontal ligament cells.

## Simple Summary

Periodontitis is a chronic inflammatory disease caused by bacterial infection, influenced by various mediators like cytokines and adipokines. This study explores the effect of adiponectin, an adipokine involved in metabolic and immune processes, on the expression of certain inflammatory cytokines like TNF-α, IL-1, IL-6, IL-8, IL-10, IL-17, and IL-18 in human periodontal ligament cells (hPDLCs). The results suggest that adiponectin enhances the expression of specific cytokines in periodontal ligament cells, which may have implications for understanding periodontitis’ pathogenesis.

## 1. Introduction

Periodontitis is a disease caused by a bacterial infection in the periodontal tissues. Bacterial infections result in chronic inflammation, the pathogenesis of which is mediated by various factors, including chemokines, cytokines, metalloproteinases, and adipokines [1]. The immune response increases the production of inflammatory mediators by immune cells and periodontal tissue cells, leading to periodontal tissue damage, including periodontal ligaments, alveolar bone resorption, and tooth loss [2]. The periodontal tissues, which include the alveolar bone, periodontal ligaments, and gums, form the apparatus that attaches the teeth to the bone. The periodontal ligament is a very important part of the periodontal tissues that connect the tooth tissue to the alveolar bone [3]. It is also involved in periodontal tissue regeneration processes. Chronic inflammation has been shown to impair the regenerative functions of periodontal tissues, leading to periodontal tissue damage. Factors involved in the development of periodontal diseases are currently being searched for, which would help to better understand the pathogenesis of this disease as well as be helpful in its treatment [4]. Population-based studies have shown a higher prevalence of periodontal disease in obese individuals [5]. It has also been shown that weight reduction can be a factor in treating periodontal diseases [6]. It has also been shown that, in addition to numerous cytokines and chemokines, adipokines secreted by adipose tissue, which may be the source of numerous mediators involved in the development of the immune response, may play a role in the development of periodontal disease [6]. One of the most important adipokines with multidirectional effects is adiponectin. Adiponectin is an adipokine secreted initially by the adipose tissue, affecting tissues through its receptors, of which three types have been identified [7,8]. Expression of receptors for adiponectin has also been found in periodontal tissues, including the periodontal ligament. The studies suggest that the expression of receptors for adiponectin in the periodontal tissues of patients with periodontitis is reduced compared to healthy individuals [9].

Adiponectin is a multidirectional adipokine with both anti-inflammatory and pro-inflammatory effects. Adiponectin influences many metabolic processes, as well as numerous immunological processes. This adipokine has been shown to have a protective effect against the development of cardiovascular disease [10]. Lower plasma adiponectin concentrations were associated with a higher risk of developing cardiovascular disease. However, it has been shown that adiponectin may have a pro-inflammatory effect in some diseases. Such effects have been shown, for example, in rheumatoid arthritis (RA), chronic kidney disease and inflammatory bowel disease [11,12]. In rheumatoid arthritis adiponectin can enhance the production of pro-inflammatory cytokines and the activity of the disease process. It has been shown that adipokines can modulate the course of both RA and periodontal disease [13]. There are many studies suggesting a link between RA and periodontal disease, as well as suggesting the involvement of similar pathogenic factors in the development of both diseases [14]. It has been shown that adiponectin may affect many processes involved in periodontitis pathogenesis, such as bone metabolism and production of cytokines [15]. Adiponectin may also influence the inflammatory status in periodontal tissues and alveolar bone resorption. The action of adiponectin likely depends on the tissues on which it acts, its concentration, the time of exposure, and the influence of other factors, such as other cytokines and chemokines. Recent studies have shown that adiponectin may play a role in modulating immune and inflammatory responses, exhibiting both anti-inflammatory and pro-inflammatory effects and thus contributing to the development of many diseases [16].

A number of complex immunological processes are involved in the pathogenesis of periodontitis, which exacerbates chronic inflammation in periodontal tissues. The ongoing inflammatory process deepens the alveolar pockets and destroys the alveolar process, causing loss of attachment [17]. It has been shown that adiponectin may also be involved in these processes [18]. Studies have shown that the periodontal ligament plays an important role in the development of periodontitis and periodontal tissue regeneration processes [3]. To date, studies have not precisely defined the role of adiponectin in the development of periodontal diseases. Some studies show an anti-inflammatory and protective role for adiponectin in the development of periodontitis, while others suggest a pro-inflammatory role for adiponectin and an exacerbation of periodontal disease [19,20,21]. In this study, we investigated the effect of adiponectin on the expression in the periodontal ligament of selected cytokines involved in the pathogenesis of periodontitis.

## 2. Materials and Methods

### 2.1. Isolation and Culture of Human Periodontal Ligament Cells In Vitro

To output the primary periodontal ligament cell line, the periodontal ligament tissue samples were collected from four healthy patients (without periodontitis) during dental surgery for orthodontic reasons and immediately transported to the laboratory in a sterile saline solution. The study was approved by the ethics committee at Pomeranian Medical University, Szczecin, Poland (KB-0012/134/18), and performed in accordance with the Declaration of Helsinki. The patients were informed about the study, and their written consent was obtained. After washed with PBS (EURx), the tissues were minced into small pieces and digested for 120 min at 37 °C with a solution of collagenase I (1 mg/mL) and collagenase II (0.5 mg/mL) (Sigma-Aldrich, St. Louis, MO, USA) with PBS. After filtered (70 µm Falcon cell strainer), cells were centrifuged in PBS (37 °C) for 8 min at 1200 rpm at room temperature (RT). Human periodontal ligament pellet cells (hPDLCs) were next resuspended in 10 mL of Dulbecco’s Modified Eagle Medium (DMEM) (Sigma-Aldrich (St. Louis, MO, USA) culture media with 10% of fetal bovine serum (FBS) (Gibco, Paisley, UK), amphotericin B (5 μg/mL), streptomycin (0.2 mg/mL), and penicillin (200 U/mL), and transferred to a 75 cm3 flask, at an initial cell density of 2.5 × 10^4^ cells/flask. The medium was replaced every 2–3 days.

### 2.2. Cell Culture Stimulation

**Figure 1:**
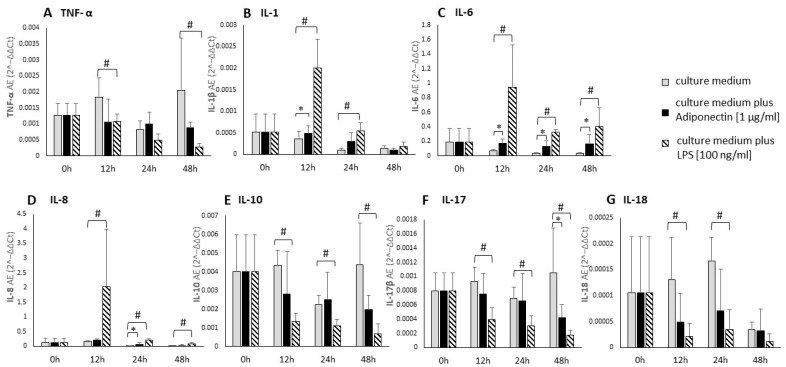
Selected cytokines mRNA expression in primary human periodontal ligament cells stimulated by adiponectin (1 µg/mL) and LPS (100 ng/mL). Effect of stimulation of primary human periodontal ligament cells with adiponectin and LPS as a positive control, on selected (**A**–**G**) pro-inflammatory cytokines. mRNA expression calculated by 2−ΔΔCt method and normalized to β2-microglobulin. Means from four repetitions ±95% confidence intervals are presented. The * sign indicates statistically significant differences between unstimulated and adiponectin-stimulated cells, where *p* < 0.05. The # sign indicates statistically significant differences between unstimulated and LPS-stimulated cells, where *p* < 0.05. (Kruskal–Wallis Test in comparison to non-stimulated control).

**Figure 2:**
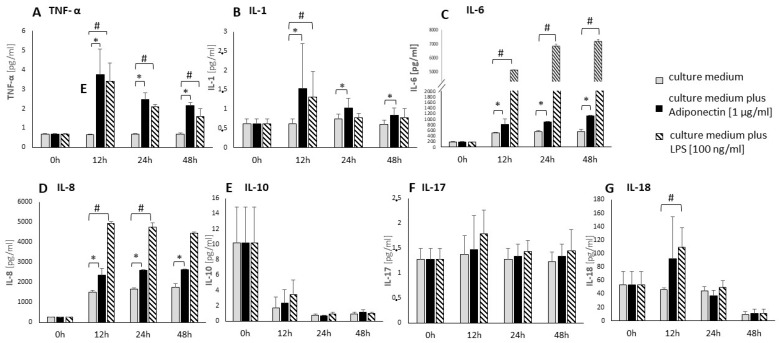
Cytokines expression in supernatant media from primary human periodontal ligament cells stimulated by adiponectin (1 µg/mL) and LPS (100 ng/mL). Effect of stimulation of primary human periodontal ligament cells with adiponectin and LPS as a positive control, on selected (**A**–**G**) pro-inflammatory cytokines. Protein concentration expressed in [pg/mL]. Means from four repetitions ±95% confidence intervals are presented. The * sign indicates statistically significant differences between unstimulated and adiponectin-stimulated cells, where * *p* < 0.05. The # sign indicates statistically significant differences between unstimulated and LPS-stimulated cells, where # *p* < 0.05. (Kruskal–Wallis Test in comparison to non-stimulated control).

Cells were harvested from the 75 cm^3^ culture flasks by trypsinisation. After centrifuging at 1200 rpm for 8 min at RT, the number of cells in the pellet was determined by a Bürker hemocytometer. The cells were seeded in 24-well plates at a density of 0.04 × 10^6^ cells per well in 300 µL medium and incubated for 24 h. The next day, the cells were washed with PBS (EURx) and the medium was replaced by new with selected stimulating factors. The hPDLCs cells were stimulated for 0, 12, 24, and 48 h with lipopolysaccharide (LPS) (100 ng/mL) or adiponectin (1 µg/mL) and the results are shown in Figure 1 and Figure 2. The “culture medium” results refers to cells with pure culture medium (not stimulated by adiponectin or LPS), the “culture medium with Adiponectin” group refers to cells cultured in medium with 1 µg/mL adiponectin (but without LPS), and the results shown in the graphs as “culture medium with LPS” refers to cells cultured in medium with 100 ng/mL LPS (without adiponectin). LPS was derived from *E. coli* (O55:B5) and purchased from Sigma-Aldrich (St. Louis, MO, USA) and adiponectin from Peprotech (Thermo Fisher Scientific Inc., Cranbury, NJ, USA)). DMEM with 10% FBS was used as a negative control. Experiments were carried out in four replicates. At each time point, the supernatants from wells were collected for Luminex assay, and cells were resuspended in 350 µL of RLT lysis buffer from RNeasy^®^ Mini Kit (Qiagen, Hilden, Germany). The cell samples were stored at −20 °C for further mRNA isolation procedures.

### 2.3. mRNA Isolation and Reverse-Transcription Polymerase Chain Reaction (PCR)

The mRNA from hPDLCs was isolated by using an RNeasy^®^ Mini Kit (Qiagen, Hilden, Germany) according to the manufacturer’s protocol. Then, the isolated mRNA was reverse-transcribed using a cDNA synthesis kit (RevertAid RT Kit, Thermo Scientific, Waltham, MA, USA) according to the manufacturer’s protocol.

### 2.4. Real-Time Quantitative Reverse Transcription PCR (qRT-PCR)

Quantitative expression analysis of the selected genes, as well as the reference gene (β2-M), was performed using real-time qRT-PCR on an ABI PRISM^®^ Fast 7500 Sequence Detection System (Applied Biosystems, Waltham, MA, USA) with Power SYBR-Green PCR Master mix reagent and specific primers (listed in Table 1). Real-time conditions were as follows: 95 °C (15 s), 40 cycles at 95 °C (15 s), and 60 °C (1 min). According to the melting point analysis, only one PCR product was amplified under these conditions. Each sample was analyzed in two technical replicates, and mean Ct values were used for further analysis. To calculate the values, the 2−ΔΔCt method was used. The values were normalized to β2-microglobulin.

### 2.5. The Assessment of Cytokines in Cell Culture Supernatant

The seven cytokine expressions were tested in each sample by a Luminex assay according to the manufacturer’s procedure (Luminex Human Discovery Assay, R&D Minneapolis, MN, USA): interleukin (IL)-1, IL-6, IL-8, IL-10, IL-17, IL-18, and tumor necrosis factor-alpha (TNF-α).

### 2.6. Statistical Analysis

Expressions and concentrations of cytokines were compared between experiments with different stimulation conditions using the Kruskal–Wallis test followed by the Mann–Whitney test if significant. * *p* < 0.05 and ** *p* < 0.005 were considered as statistically significant.

## 3. Results

### 3.1. The Effect of Adiponektin on the TNF-α, IL-1, IL-6, IL-8, IL-10, IL-17, IL-18 mRNA Expresion in Periodontal Ligament Cells

In the first part of the study, we examined the effect of adiponectin on the expression in periodontal ligament cells of mRNA of mediators involved in periodontitis pathogenesis (*TNF-α*, *IL-1*, *IL-6*, *IL-8*, *IL-10*, *IL-17*, *IL-18*). The samples were analyzed after 12, 24, and 48 h of stimulation with adiponectin (1 µg/mL), and presented below in Figure 1. We found no significant effect of adipokine on *TNF-α* gene expression after 12, 24, and 48 h of stimulation. For IL-1, a statistically significant increase in *IL-1* gene expression was found after 12 h of adiponectin stimulation, while the differences were not statistically significant after 24 and 48 h. Adiponectin caused a statistically significant increase in *IL-6* gene expression after 12, 24, and 48 h of stimulation (Figure 1C). For *IL-8*, a statistically significant increase in its gene expression was observed after 12 h of adiponectin stimulation. There was no statistically significant effect of adiponectin on *IL-10* gene expression. Adiponectin caused a significant decrease in the *IL-17* gene expression after 48 h of stimulation. There was no statistically significant effect of adiponectin on *IL-18* gene expression (Figure 1G).

### 3.2. The Effect of Adiponectin on TNF-α, IL-1, IL-6, IL-8, IL-10, IL-17, and IL-18 Protein Levels in Supernatants of Periodontal Ligament Cells Cultures

The second stage of the study investigated the effect of adiponectin on TNF-α, IL-1, IL-6, IL-8, IL-10, IL-17, and IL-18 protein levels in supernatants of periodontal ligament cell cultures. The samples were analyzed after 12, 24, and 48 h of stimulation with adiponectin (1 µg/mL), and presented in Figure 2. Stimulation of periodontal ligament cells with adiponectin significantly increased TNF-α, IL-6, and IL-8 protein levels after 12, 24, and 48 h (Figure 2A,C,D). IL-1 levels statistically significantly increased after 12 and 24 h of adiponectin stimulation (Figure 2B). There was no statistically significant effect of adiponectin on IL-10, IL-17 and IL-18 levels (Figure 2E–G).

## 4. Discussion

This study aimed to examine the effect of adiponectin on the expression in the periodontal ligament of cytokines involved in the pathogenesis of periodontal disease. We assessed the impact on gene expression of these cytokines in the periodontal ligament and their protein concentrations in culture supernatants of periodontal ligament cells. We showed a statistically significant effect of adiponectin stimulation on TNF-α protein levels in cell culture supernatants after 12, 24, and 48 h of adiponectin stimulation. In contrast, there was no effect of adiponectin stimulation on TNF-α gene expression after 12, 24, and 48 h of adiponectin stimulation. This surprising fact can be explained by earlier observations, in which it was shown that after LPS stimulation, TNF-α gene expression temporarily increases up to four hours and then decreases [22]. The increased TNF-α concentrations we observed after 12, 24, and 48 h of adiponectin stimulation are probably due to the increase in temporary *TNF-α* gene expression, which caused the rise in TNF-α protein synthesis that was noted up to 48 h. In the case of IL-1, an increase in its gene expression was observed after 12 and 24 h of adiponectin stimulation. On the other hand, increased IL-1 protein levels persisted even after 48 h of stimulation. In the case of IL-6, adiponectin stimulation caused a statistically significant increase in both *IL-6* gene expression and protein levels in cell culture supernatants of the periodontal ligament cells after 12, 24, and 48 h of stimulation. IL-6 is a pro-inflammatory cytokine involved in the pathogenesis of periodontitis. Patients with periodontitis have been found to have elevated levels of IL-6 in gingival fluid and serum [23]. IL-6 affects the differentiation of B and T lymphocytes, thereby increasing inflammation in periodontal tissues [24]. IL-6 also increases osteoclast differentiation, leading to destruction of the alveolar process [25]. Previous studies have shown that adiponectin can increase IL-6 production in human synovial membrane fibroblasts via the AdipoR1 receptor, AMPK, p38 and the NF-kappa B pathway [26].

In the case of IL-8, there was an increase in its protein level in cell culture supernatants after 12, 24, and 48 h of adiponectin stimulation. However, a statistically significant increase in its gene expression was found after 24 h of stimulation. This result, similar to that of TNF-α, can be explained by an earlier increase in *IL-8* gene expression before 12 h, which may have subsequently decreased and increased again after 24 h of stimulation. These changes in IL-8 expression resulted in increased protein levels in culture supernatants observed at 12, 24, and 48 h. There was no statistically significant effect of adiponectin on the gene and protein expression of IL-10 and IL-18. IL-10 is an anti-inflammatory cytokine that can reduce the inflammatory response in patients with periodontitis [27]. It has been shown that IL-10 can inhibit osteoclast activity and prevent the destruction of the alveolar process [28]. In addition, IL-10 reduces the expression of pro-inflammatory cytokines and the proliferation of Th17 cells [29]. Upregulation of IL-10 expression inhibits the proliferation of human periodontal ligament stem cells [30]. Studies conducted on macrophages and T lymphocytes have shown an increase in IL-10 expression under the influence of adiponectin [31,32].

In our study, we observed that *IL-17* gene expression decreased after 48 h of adiponectin stimulation, which has not yet been observed at the protein level. IL-17 is a pro-inflammatory cytokine that enhances the development of periodontitis. IL-17 activates osteoclasts and RANKL, which directly causes bone destruction [33]. Elevated levels of IL-17 have been found in the gingival fluid of patients with periodontitis [34]. It has been shown that adiponectin reduces the production of IFN-γ and IL-17 by CD4 T cells in obesity by reducing intracellular glycolysis [35]. Also, in models of psoriasis, adiponectin reduced IL-17 expression [36]. Shibata et al. have shown that adiponectin directly acts on murine dermal γδ-T cells to suppress IL-17 synthesis via AdipoR1 [37]. In our study, there was no effect of adiponectin on IL-17 protein expression within 48 h of stimulation. However, the reduction in *IL-17* gene expression after 48 h of adiponectin stimulation may suggest that prolonged adiponectin stimulation could probably also reduce IL-17 protein expression in periodontal ligament cells.

The direction of action of adiponectin may depend on the cells on which it acts, the microenvironment, the timing of its exposure, and the interaction of other cytokines and chemokines. The periodontal ligament has been shown to play an important role in the development of periodontal disease, both as a source of inflammatory mediators and important regenerative functions [3]. *Porphyromonas gingivalis* and LPS have been shown to increase the expression of many pro-inflammatory cytokines and chemokines in periodontal ligament cells that are involved in the development of inflammation [38,39]. Several mediators act on the cells of the periodontal ligament, thus exacerbating the inflammation and causing periodontal tissue inflammation [40]. Previous studies have shown an association between periodontal disease and obesity, which may suggest a role for adipokines in the pathogenesis of periodontitis. In addition, weight reduction is beneficial in treating periodontal disease [7,8].

To date, many studies have assessed the role of adiponectin in the pathogenesis of periodontal disease, but these have been inconclusive [15]. Research suggests that adiponectin has both a pro-inflammatory effect on periodontal tissue and an anti-inflammatory effect and may have a positive impact on periodontal tissue regeneration. Studies in animal models have shown that blocking adiponectin receptors can counteract the development of periodontitis [20]. Blocking the adiponectin receptor has been shown to reduce alveolar bone resorption and gingival inflammation in rats with periodontitis. Inhibiting the adiponectin receptor decreased monocyte and macrophage migration by reducing CCL2 secretion. In addition, nuclear factor kappa-light-chain-enhancer of activated B cells (NF-κB) expression was observed [20]. Wu et al. showed that blocking the adiponectin receptor inhibited osteoclastogenesis. In mice with experimental periodontitis, adiponectin receptor blockade counteracted alveolar bone destruction, reduced the number of osteoclasts, and decreased the expression of pro-inflammatory mediators in periodontal tissues [41]. There are also many studies suggesting an anti-inflammatory effect of adiponectin in periodontal diseases. Park et al. showed that adiponectin initially increases TNF-α expression but then increases the synthesis of other factors that may have anti-inflammatory effects [42]. Furthermore, it has been shown that adiponectin can inhibit the inflammatory process by enhancing autophagy in macrophages [43]. Furthermore, adiponectin can increase the expression of other anti-inflammatory factors, such as transforming growth factor beta-1 (TGF-β1) and enhance collagen synthesis [44,45,46,47]. In another study, adiponectin increased the expression of growth factors and extracellular matrix as well as the proliferation of periodontal ligament cells in vitro [48].

Wu et al. demonstrated the anti-inflammatory action of adiponectin in human periodontal ligament cells and enhanced osteogenesis [49]. One of the major symptoms of periodontal disease is the destruction of the alveolar process. However, the effect of adiponectin on bone metabolism has not been fully elucidated. Most researchers conclude that adiponectin has a rather protective effect against bone degradation. Animal studies suggest a positive effect of adiponectin on bone formation. Studies suggest that adiponectin enhances the differentiation of osteoblast progenitor cells and inhibits the differentiation of osteoclast progenitor cells, thereby enhancing bone formation on the one hand and inhibiting bone destruction on the other. Moreover, it has been shown that adiponectin can enhance bone mineralization processes [50,51]. In addition to the effect of adiponectin on bone tissue, studies suggest its effect on collagen synthesis and cross-linking, which may result in the strengthening of the periodontal ligaments [52,53].

Previous studies have shown an association between obesity and adipokines and the development of periodontitis [6,54]. Obese patients have been shown to have an increase in T lymphocytes and macrophages, which are the source of many of the cytokines responsible for inflammation in patients with periodontitis. A high-fat diet has also been shown to increase the secretion of pro-secretory cytokines by immune cells [54]. It has been shown that adiponectin can modulate macrophage function in experimental periodontitis [55]. In another study, it was shown that adiponectin can increase inflammation through neutrophil activation. Adiponectin increased the synthesis of pro-inflammatory cytokines (TNF-α, IL-1 and IL-6) in macrophages and increased cyclooxygenase (Cox2) expression [12].

The results of previous studies and our findings suggest a very complex involvement of adiponectin in the pathogenesis of periodontal disease. Adiponectin appears to be part of a very complex cascade of processes contributing to the development of inflammation, which destroys periodontal tissues. Previous studies indicate that the action of adiponectin is multidirectional, depending on the tissues on which it acts, the time of its action, and its interaction with other mediators involved in the development of inflammation [56,57,58]. It has been shown that *Porphyromonas gingivalis* can induce an inflammatory response and inhibit adiponectin synthesis in periodontal tissues, which may be one of the mechanisms through which this bacterium influences the development of periodontitis [59]. Our results showed that adiponectin may increase the expression of selected pro-inflammatory mediators involved in the development of periodontitis, but this increase may be temporary. Therefore, it is difficult to assess what long-term effect this has on the development of periodontitis. Increased expression of some pro-inflammatory cytokines does not predetermine adiponectin’s pro-inflammatory effect in periodontal disease, as adiponectin can influence a number of pathways and processes, including anti-inflammatory pathways and inhibition of bone degradation. The ultimate impact of adiponectin on periodontal tissues and the development of periodontitis depends on which pathways are activated by its action.

## 5. Conclusions

Adiponectin may significantly increase the expression of selected cytokines in periodontal ligament cells. Our results seem to confirm the multidirectional effect of adiponectin in periodontitis. The final effect probably depends on the influence of many other factors, such as bacterial infections or other mediators that influence the development of inflammation. However, assessing the role of adiponectin in the pathogenesis of periodontal disease seems justified and requires further research.

## Figures and Tables

**Table 1:** Primer sequences used in the experiment.

Gen	Forward Sequence	Reverse Sequence
β2-M	5′-AATGCGGCATCTTCAAACCT-3′	5′-TGACTTTGTCACAGCCCAAGA-3′
TNF-α	5′-GATGATCTGACTGCCTGGGC-3′	5′-CACGCTCTTCTGCCTGCTG-3′
IL-1α	5′-ACAGATGAAGTGCTCCTTCCA-3′	5′-GTCGGAGATTCGTAGCTGGAT-3′
IL-6	5′-CACTGGTCTTTTGGAGTTTGAG-3′	5′-GGACTTTTGTACTCATCTGCAC-3′
IL-8	5′-AACCCTCTGCACCCAGTTTTC-3′	5′-ACTGAGAGTGATTGAGAGTGGAC-3′
IL-10	5′-GGTTGCCAAGCCTTGTCTGA-3′	5′-AGGGAGTTCACATGCGCCT-3′
IL-17α	5′-GAGCCCCAAAAGCAAGAGGAA-3′	5′-TGCGGGCATACGGTTTCATC-3′
IL-18	5′-ATCGCTTCCTCTCGCAACAA-3′	5′-CTTCTACTGGTTCAGCAGCCATCT-3′

Abbreviations: β2-M, beta-2 microglobulin; TNF-α, tumor necrosis factor alpha; IL, interleukin.

## Data Availability

Data are contained within the article.

## Abbreviations

The following abbreviations are used in this manuscript:

AdipoR1	Adiponectin receptor 1
ADP	Adiponectin
AMPK	5′AMP-activated protein kinase
Cox2	Cyclooxygenase
hPDLC	Human Periodontal Ligament Cells
IL	Interleukin
IFN-γ	Interferon gamma
LPS	Lipopolysaccharides
NF-κB	Nuclear Factor Kappa-Light-Chain-Enhancer of Activated B Cells
RA	Rheumatoid Arthritis
RANKL	Receptor Activator for Nuclear Factor κ B Ligand
TNF-α	Tumor Necrosis Factor Alpha
TGF-β1	Transforming Growth Factor Beta-1
